# Maternal-Perinatal Variables in Patients with Severe Preeclampsia Who Develop Acute Kidney Injury

**DOI:** 10.3390/jcm10235629

**Published:** 2021-11-29

**Authors:** Patrocinio Rodríguez-Benitez, Irene Aracil Moreno, Cristina Oliver Barrecheguren, Yolanda Cuñarro López, Fátima Yllana, Pilar Pintado Recarte, Coral Bravo Arribas, Melchor Álvarez-Mon, Miguel A. Ortega, Juan A. De Leon-Luis

**Affiliations:** 1 Department of Public and Maternal and Child Health, School of Medicine, Complutense University of Madrid, 28040 Madrid, Spain; prodriguezb@senefro.org (P.R.-B.); irene.aracil@salud.madrid.org (I.A.M.); cristina.oliver@salud.madrid.org (C.O.B.); yolanda.cunarro@gmail.com (Y.C.L.); lourdesfatima.yllana@salud.madrid.org (F.Y.); ppintado@salud.madrid.org (P.P.R.); cbravoarribas@gmail.com (C.B.A.); jaleon@ucm.es (J.A.D.L.-L.); 2 Department of Obstetrics and Gynecology, University Hospital Gregorio Marañón, 28009 Madrid, Spain; 3 Health Research Institute Gregorio Marañón, 28009 Madrid, Spain; 4 Department of Nephrology, University Hospital Gregorio Marañón, 28009 Madrid, Spain; 5 Department of Medicine and Medical Specialities, University of Alcala, 28801 Alcala de Henares, Spain; mademons@gmail.com; 6 Ramón y Cajal Institute of Sanitary Research (IRYCIS), 28034 Madrid, Spain; 7 Immune System Diseases-Rheumatology, Oncology Service an Internal Medicine, University Hospital Príncipe de Asturias, (CIBEREHD), 28806 Alcala de Henares, Spain

**Keywords:** kidney injury, preeclampsia, chronic kidney disease, microangiopathy, maternal-perinatal

## Abstract

**Introduction:** At present, we are witnessing an increase in preeclampsia, especially the most severe forms, which are associated with an increased risk of maternal-perinatal morbidity and mortality. As a severity criterion, acute kidney injury (AKI) has been associated with a worse prognosis, and for this reason, the maternal and perinatal variables associated with AKI in patients with severe preeclampsia (SP) were analysed in this study. **Methods:** An observational, retrospective, single-centre study of patients with SP treated at a tertiary hospital between January 2007 and December 2018 was conducted. The case criteria based on the criteria established by the ACOG Practice Guidelines for Gestational Hypertension and Preeclampsia. AKI is considered when serum creatinine exceeds 1.1 mg/dL in a pregnant woman with previously normal renal function. In patients with existing chronic kidney disease (CKD), it is referred to as AKI if the baseline serum creatinine increases by 1.5 fold. Pregestational, gestational and postpartum variables were analysed up to 12 weeks postpartum using univariate and multivariate logistic regression analysis. **Results:** During the study period, 76,828 births were attended, and 303 pregnant women were diagnosed with SP. The annual incidence of SP increased gradually throughout the study period, reaching 1.79/100 births/year in 2018. Acute kidney injury (AKI) occurred in 24.8% of the patients. The multivariate analysis revealed an increased association with a history of previous CKD, the use of assisted reproductive techniques and caesarean section. Uric acid and thrombotic microangiopathy (TMA) had a high correlation with AKI. Indications for caesarean section are associated with AKI in SP. Regarding perinatal outcomes in cases of AKI, there was a higher percentage of neonates who required foetal lung maturation with steroids and an increased need for NICU admission. No case of maternal death was recorded; however, an increase in neonatal mortality was found among patients who did not develop AKI. After 12 weeks postpartum, 72 patients were referred to the nephrology consultation for persistent hypertension, proteinuria or renal failure. **Conclusions:** In preeclampsia, AKI is a common complication, especially among patients with a history of CKD, those who became pregnant using assisted reproduction techniques and those who delivered via caesarean section. The perinatal impact of AKI is mainly centred on a higher rate of NICU admission and a lower mortality rate. Among biochemical and haematological markers, the uric acid level prior to renal failure has a direct and significant correlation with the risk of AKI, as does the development of TMA in patients with preeclampsia. Therefore, the monitoring of renal function in cases of preeclampsia should be strict, and referral for a nephrology consultation may be necessary in some cases.

## 1. Introduction

Preeclampsia (PE) is a syndrome characterised by the de novo appearance in a woman more than 20 weeks pregnant of hypertension (HT) associated with proteinuria and/or other manifestations of organ dysfunction. It is included within the category of hypertensive disorders of pregnancy (HDP), a term that refers to a set of entities whose link is the presence of arterial HT in a pregnant woman [1,2].

There are clinical and analytical conditions that confer severity upon preeclampsia and further increase the risk of maternal-perinatal morbidity and mortality [3,4]. Among them are systolic blood pressure ≥ 160 mmHg and/or diastolic blood pressure ≥ 110 mmHg; proteinuria ≥ 2 g; oliguria; renal failure; neurological or visual alterations; acute pulmonary oedema or cyanosis; pain in the epigastrium or right hypochondrium; liver dysfunction; haematological alterations or placental involvement, with foetal manifestations such as delayed intrauterine growth.

At present, we are witnessing an increase in the incidence of preeclampsia and SP; it is a frequently encountered obstetric complication and one of the main causes of maternal-perinatal morbidity and mortality [5,6,7,8,9,10,11]. In pregnancy, the glomerular filtration rate (GFR) increases by 40–60% compared to normal levels, which leads to a decrease in plasma creatinine levels (normal levels in pregnant women are 0.4–0.6 mg/dL) [12,13,14]. The formulas used to estimate the GFR with creatinine are not validated for pregnancy, which makes the diagnosis of acute kidney injury (AKI) difficult in this clinical situation [13]. As a result of these factors, there is controversy regarding the definition of AKI during pregnancy, which has led to different ways of defining it in the literature [14,15]. AKI, generally defined as a decrease in the GFR within hours or days, is a frequent complication in the hospital setting, especially in cases of previous chronic kidney disease (CKD) and chronic renal failure (CRF), defined as GFR < 60 mL/min/1.73 m^2^ It is classically defined as a rare complication within the maternal clinical manifestations of PE. When it occurs in this context, it is associated with a worse maternal and perinatal prognosis [16]. In fact, as previously mentioned, it is one of the clinical criteria that defines severe preclampsia (SP) and necessitates important considerations, such as whether to terminate the pregnancy, as it has a decisive influence on maternal and perinatal prognoses [3,4].

The objective of this study is to compare the maternal and perinatal clinical and analytical variables in patients with SP who develop AKI vs. those who do not develop this condition.

## 2. Materials and Methods

Design and patients: An observational study was conducted with a retrospective, hospital-based cohort of patients with SP treated at a tertiary centre between January 2007 and December 2018 to compare maternal-perinatal variables between patients who develop AKI and those who do not. SP was diagnosed according to the criteria of the American College of Obstetricians and Gynecologists Practice Guidelines for Gestational Hypertension and Preeclampsia [16] in patients with preeclampsia who met some of the following severity criteria: Systolic blood pressure (SBP) ≥ 160 mmHg and/or Diastolic blood pressure (DBP) ≥ 110 mmHg confirmed at 15 min; proteinuria ≥ 2 g measured in 24-h urine or estimated by the protein/creatinine ratio in urine; oliguria ≤ 500 mL/24 h or diuresis rate < 0.5 mL/kg/h for 2 h; renal insufficiency: serum creatinine > 1.1 mg/dL, or double the value of serum creatinine in the absence of other renal disease; neurological or visual alterations, including severe headache that does not subside with analgesics, blurred vision, diplopia or amaurosis; acute lung oedema or cyanosis; pain in the epigastrium or right hypochondrium; hepatic dysfunction: transaminase levels elevated to double the normal value; haematological alterations, including thrombocytopenia (<100,000 mm^3^), disseminated intravascular coagulation (DIC) or haemolysis; placental involvement with foetal manifestations including intrauterine growth restriction (IGR), abnormal umbilical artery Doppler results and foetal death [3,4]. In this study, we considered the presence of a serum creatinine level greater than 1.1 mg/dL, which is a criterion for the severity of preeclampsia [16]. This cut-off point takes into account the Kidney Disease: Improving Global Outcomes (KDIGO) definition and attempts to avoid overestimating the incidence of AKI by including mild forms of renal function impairment.

In patients with pre-existing CKD, AKI was considered when the baseline serum creatinine level was 1.5 times higher than the baseline level. Oliguria was considered at a diuresis rate of less than 500 mL in 24 h or less than 0.5 mL/kg/h for 2 consecutive hours [16]. If AKI was accompanied by oliguria, it was defined as oliguric AKI. CKD was defined according to the criteria of the National Kidney Foundation-Kidney Disease Outcomes Quality Initiative (NKF-KDOQI) guidelines [17]. Also for CKD we included patients with proteinuria at <20 weeks HELLP (H: hemolysis, EL: elevated liver enzymes and LP: low platelets) syndrome was diagnosed according to the Sibai criteria: haemolysis (schistocytes in smear, LDH ≥ 600 IU/L or bilirubin ≥ 1.2 mg/dL), platelet count < 100,000 cells/µL and GPT ≥ 70 U/L [18]. Thrombotic microangiopathy (TMA) was clinically defined by the presence of microangiopathic haemolytic anaemia, thrombocytopenia and organ dysfunction, with primary involvement of the kidneys [19].

The study protocol was approved by the centres Ethics Committee for Medical Research, and patient follow-up was performed from the point at which the women were diagnosed with SP during either pregnancy, childbirth or postpartum, mainly until the normalisation of blood pressure (without the need for antihypertensive medication) and proteinuria; the maximum postpartum follow-up duration was 12 weeks in cases of specialised postpartum consultation for at-risk patients.

The study variables were collected at the following time points: pre-gestational; gestational; at the time of the preeclampsia diagnosis; peripartum; perinatal; upon discharge from the hospital; and at the 12-weeks postpartum consultation.

The study data were stored in a database created for this purpose until the statistical analysis. The variables were analysed using the statistical package IBM Corp. Released 2015. IBM SPSS Statistics for Windows, Version 23.0. Armonk, NY: IBM Corp. are presented as means and SDs for quantitative variables and as number and percentages for qualitative variables. For the comparison of the variables between the study groups, we conducted a univariate analysis by logistic regression to determine the probability ratio, odds ratio (OR) and 95% confidence interval (CI). After univariate analysis, variables with clinical relevance or a *p*-value equal to or less than 0.20 were included in the multivariate logistic regression analysis. Results with *p* less than 0.05 were accepted as significant.

## 3. Results

During the study period, 76,828 births took place at the centre; of these, 303 were to women diagnosed with SP. The annual incidence increased gradually over the study period, reaching 1.79% births/year in 2018. During the study period, no cases of maternal death were recorded among patients with SP. AKI occurred in 75 patients (24.8%), of whom 34.66% had oliguria, with a mean serum creatinine of 1.53 ± 0.73 mg/dL and a mean urea of 58.39 ± 25.83 mg/dL. In 32% of cases, AKI was caused by CKD, and in 5.33% (4 patients), it was caused by CRF. In the AKI group, 25.33% (19 patients) developed AKI in the context of HELLP syndrome, and in 3 developed AKI in the context of haemolytic uraemic syndrome secondary to pregnancy. Twenty-one patients who developed AKI (28%) required the transfusion of packed red blood cells; 12 of these patients also had HELLP syndrome, and 3 also had haemolytic uraemic syndrome secondary to pregnancy. Only 1 patient required temporary dialysis to recover renal function.

Table 1, Table 2, Table 3, Table 4, Table 5, Table 6 and Table 7 summarise the descriptive statistics of the study variables for all patients diagnosed with SP (*n* = 303), those who developed AKI (*n* = 75) and those who did not develop AKI (*n* = 228). As Table 1 shows, the comparative analysis found significant differences in personal history of CKD, CRF, mean baseline serum creatinine and mean pregestational proteinuria. Table 2 show that in AKI patients there were a significantly higher percentage of patients who became pregnant through in vitro fertilisation (IVF) and patients who had proteinuria before 20 weeks of gestation.

Regarding the variables measured at the time of SP diagnosis, presented in Table 3, the occurrence of AKI was positive associated with the gestational week at which SP was diagnosed, OR 1.67 (CI 95%: 1.07–2.61). Statistically significant differences were found in the mean levels of some biochemical variables in maternal blood. The difference in uric acid was especially notable, with an OR 2.11 (CI 95%: 1.70–2.60) in AKI group. The mean levels of platelets, haemoglobin and albumin were significantly lower in the AKI group, as were the plasma levels of C3. The AKI group had a significantly higher percentage of patients with HELLP, and patients treated with diuretics and magnesium sulfate than the group without AKI.

Regarding variables measured intrapartum and immediately postpartum, shown in Table 4, we found a significantly higher percentage of patients in the AKI group who had a caesarean section, OR: 3.35 (CI 95%: 1.74–6.43), or required a transfusion. Regarding the week of gestation at the time of delivery, the group with AKI had a higher percentage of premature delivery (<37 weeks of gestation) OR: 3.4 (CI 95%: 1.7–6.4). There were also significant differences in the type of antihypertensive treatment used during the immediate postpartum period: a lower percentage of patients in the AKI group were treated with enalapril, and a higher percentage were treated with calcium antagonists. No significant differences were found for the rest of the variables.

A perinatal mortality rate of 5.1% was found for all 351 neonates. A total of 64% were premature (<37 weeks of gestation), and 5.3% were extremely premature at less than 28 weeks of gestation at birth. The mean gestational age at delivery was 34 weeks. Table 5 presents the perinatal variables and shows that a significantly higher percentage of the neonates born to the AKI group required foetal lung maturation with steroids, OR 1.89 (CI 95%: 1.11–3.21) and ICU admission. The mean Apgar scores at 1 min and 5 min of life were significantly lower in the AKI group than in the non-AKI group, OR 0.74 (CI 95%: 0.61–0.91). Regarding IGR, no differences were found between the groups. In contrast, the percentage of infants who were small for their gestational age (SGA) was higher in the non-AKI group.

Regarding the variables measures at the time of hospital discharge, shown in Table 6, there was a lower percentage of hypertensive patients in the AKI group than in the no-AKI group, OR 0.15 (CI 95%: 0.05–0.44), and there were statistically significant differences in the number of antihypertensive drugs being taken at discharge. There were no differences in the percentage of patients with proteinuria at discharge, although the mean proteinuria level at discharge was significantly higher in the group with AKI. There were also significant differences in the mean GPT, uric acid, LDH and haemoglobin values at discharge.

After hospital discharge, 9.2% of the participants were lost to follow-up. At discharge from the postpartum risk consultation, as shown in Table 7, the highest percentage of patients lost to follow-up were in the no-AKI group. On the other hand, there were statistically significant differences between groups in the percentage of patients with persistent proteinuria at 12 weeks postpartum, OR 3.31 (CI 95%: 1.54–7.11), and in the mean value of proteinuria in the AKI group than in the no-AKI group.

After a maximum follow-up of 12 weeks postpartum, 25 patients (9%) without a previous history of chronic HT remained hypertensive. Similarly, 19 patients (7%) without pregestational proteinuria showed persistent postpartum proteinuria (protein/creatinine index greater than 0.3 mg/mg), and 7 patients (2.3%) had persistent AKI, including 2 who had previous CRF. After 12 weeks postpartum, 72 patients were referred to the nephrology consultation for persistent HT, proteinuria or renal failure.

**Figure 1:**
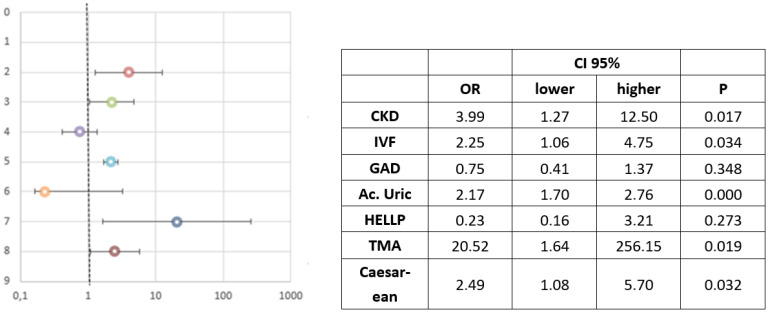
Association study of maternal-perinatal variables with acute renal failure in the group of pregnant women with severe pre-eclampsia. Multivariate logistic regression analysis (variables included in the model). Pre-pregnancy CKD; IVF in vitro fertilization; GAD gestational age at diagnosis of AKI; Ac. Uric pre AKI; HELLP syndrome; TMA thrombotic micro angiopathy; indication for caesarean section.

In the multivariate logistic regression analysis (Figure 1), previous CKD and IVF predicted an increased risk of AKI onset. The pre-AKI uric acid level was the strongest biochemical marker of the onset of AKI. TMA was a cause of AKI in SP. Indications for caesarean section were associated with the onset of AKI in SP.

## 4. Discussion

Our results describe a high frequency of AKI in our series of pregnant women with SP stands out: it affects 25% of these patients and 9.76/10,000 births in our hospital-based population. In SP cases, AKI occur in the third trimester and in the immediate postpartum period, and HDP, mainly preeclampsia and HELLP syndrome, are the main causes [13,20,21]. Other maternal and perinatal variables associated with AKI cases were a history of CRF, pregnancies after assisted reproductive techniques use, higher maternal blood of uric acid and creatinine levels, higher rate of preterm delivery, c-section and postpartum complications such as haemorrhage the need for red blood cell transfusion. Despite a higher rate of preterm and admission in neonatal ICU, neonatal mortality rate was significantly lower in AKI cases.

Comparing to the literature, in a series of pregnancy-associated AKI in India, the researchers observed that 17% of patients with preeclampsia and 60% of patients with HELLP syndrome developed AKI [20]. In any case, it is important to consider other causes of AKI associated with pregnancy, which, although infrequent, are very serious. Examples include forms of TMA that can occur in the final phase of pregnancy or immediately postpartum and that are often difficult to differentiate from SP or HELLP syndrome. In fact, both SP and HELLP syndrome are currently considered types of TMA [22]. Our incidence is clearly higher than that described in the literature, including the incidences of 2.68/10,000 births in a Canadian study [23] and 4.5/10,000 births in a US series [24].

Seventy-five percent of cases of AKI occur in the third trimester and in the immediate postpartum period, and HDP, mainly preeclampsia and HELLP syndrome, are the main causes [13,20,21]. Preeclampsia involves histological changes at the renal level that are characterised by glomerular endotheliosis, podocyturia and proteinuria; functional changes, such as decreased renal tubular secretion of uric acid; and haemodynamic alterations consisting of intrarenal vasoconstriction, decreased renal plasma flow and a GFR reduction of between 30 and 40% [25]. These conditions lead to susceptibility to renal ischaemic injury and the onset of AKF.

In the general population, one of the main risk factors for AKI is a history of CRF and, above all, its severity [26]. The results of our study are consistent with these data, showing an OR 12.79 (CI 95%: 1.41–116.28), *p* = 0.02, for CRF. This result is supported by the findings of other authors [27,28].

Regarding pregnancy-related variables, a higher frequency of assisted reproductive techniques and IVF use was found among the SP patients who developed AKI than in those without AKI (32 vs. 17.1%). The risk of developing AKI was 2.28 times higher among patients with SP who underwent IVF. To date, we have not found any studies that that relate AKI to the use of assisted reproductive techniques in patients with SP, although there are studies that describe the relationship between AKI and assisted reproduction techniques in pregnant women in general [29,30]. What is well established is that preeclampsia is the most frequent cause of AKI in developed countries [13,21,23,24,31,32], and it is very likely that the established relationship between preeclampsia and assisted reproductive techniques justifies our results [33,34,35,36].

When analysing the association between the development of AKI and variables measured at the time of SP diagnosis, it was found that the mean uric acid value was significantly higher in the group with SP and AKI than in the group without AKI, 8.87 ± 1.74 vs. 6.88 ± 1.45 mg/dL, *p* < 0.001. This result is explained by the fact that uric acid is eliminated mainly by the kidneys and therefore, when SP develops and the GFR and tubular secretion decrease, uric acid levels increase [37]. Hyperuricaemia is correlated with the severity of glomerular endotheliosis, and in pregnant women with SP, the level of uric acid is an early marker of kidney damage and maternal-foetal prognosis [38,39,40,41,42]. In this sense, Le TM et al. [40] found that a uric acid level of 6.6 mg/dL is a good predictor of the severity of preeclampsia/eclampsia, OR 5.19 (CI 95%: 2.79–9.65). A possible line of study is the use of this and other markers of the progression of renal damage in SP.

In our series, HELLP syndrome, a form of TMA associated with pregnancy [22,43,44], occurred significantly more frequently in the group of women with SP who developed AKI than in those who did not develop AKI (25.3% vs. 5.3%, respectively). Many studies have established the role of HELLP syndrome in AKI associated with pregnancy [15,20,21,22,44,45,46,47]. In the series by Jai Prakash et al. [21] that included 132 pregnant women with AKI, HELLP syndrome was responsible for 6.8% of all cases. This percentage is even higher in the series of Huang C et al. [47], in which HELLP syndrome was responsible for 60% of AKI cases. It is important to note that these authors defined AKI as serum creatinine levels greater than 0.8 mg/dL. In the meta-analysis of Liu Y et al. [15], in which a group of 834 patients with pregnancy-associated AKI was compared to 5334 pregnant women without AKI, the pregnant women with AKI had a 1.86-fold higher risk of having HELLP syndrome than the pregnant women without AKI. We mention this work to highlight the relevance of the temporality of the analysis of the causal association between AKI and HELLP syndrome. In our study, we considered AKI a consequence of HELLP syndrome, with an OR of 6.11 (CI 95%: 2.8–13.33), *p* < 0.01. In contrast, the aforementioned authors considered that AKI determines the risk of developing HELLP syndrome [15]. Although we demonstrated an association, we understand the clinical difficulty of determining which pathology precedes the other. The sudden onset of both clinical pictures makes it difficult to obtain definitive conclusions in one direction or another.

Regarding the measurement of variables at the time of delivery, we observed a statistically significant association between the development of AKI and gestational age at the time of delivery, with a higher percentage of preterm births (<37 weeks of gestation) to patients with AKI. In the meta-analysis of Liu Y et al. [15] mentioned above, the gestational age at the time of delivery was 0.7 weeks lower in the group of pregnant women with AKI. In our cohort of pregnant women with SP, the gestational age at the time of delivery was 1 week lower in the group of patients with AKI than in the group of patients without AKI: 33.8 vs. 34.7 weeks. This finding can be explained, at least in part, by the fact that AKI is severity criterion in pregnant women with preeclampsia and often determines the completion of childbirth [48].

The risk of caesarean section was 3.35 times higher in the SP group with AKI than in the group without AKI (CI 95%: 1.74–6.46, *p* < 0.01). This value is higher than that reported in the meta-analysis of Liu Y et al. [15], who found a 1.49 times higher risk of caesarean section in the AKI group (OR 1.49 (CI 95%: 1.37–1.61)). In this case, it is important to emphasise that our study included only women with SP, a group that was not included in the AKI group in the meta-analysis by Liu Y et al. [15]. In the study by Huang C et al. [47], the incidence of AKI in patients with SP who underwent caesarean section was as high as 60%. The clinical situation of the patient, the heterogeneity for AKI definition used by the authors and the intraoperative management of blood volume can decisively influence the incidence of AKI associated with caesarean section [49,50,51]. In our series, we relate caesarean section to the association between AKI in the SP patients however, since we did not record the time at which AKI appears in patients, before or after cesarean section, we cannot attribute a causal association of it to the development of AKI. In AKI cases after cesarean section, it could be explained that cesarean section involves greater volume loss, compared to vaginal delivery, with possible acute hemodynamic changes that lead to AKI. On the contrary, in the case in which a patient develops AKI during pregnancy and with the intention of improving the maternal-fetal prognosis, the termination of the pregnancy is decided by caesarean section due to unfavorable obstetric conditions.

There was also a statistical association between the development of AKI and the need for red blood cell transfusion, with transfused patients presenting a 9.46 times higher risk of developing AKI (OR 9.46, CI 95%: 4.10–21.83). Peripartum haemorrhage is a frequent cause of AKI associated with pregnancy and is even more common than preeclampsia in some developing countries [52,53,54]. Severe cases that require transfusion present an ischaemia-reperfusion model that can explain the development of acute tubular necrosis as a cause of AKI associated with childbirth [55].

Regarding the perinatal variables, it should first be noted that the need for steroids for foetal lung maturation was 1.89 times more frequent in the SP with AKI group OR 1.89 (CI 95%: 1.1–3.21, *p* < 0.05). The higher need for treatments for foetal lung maturation is probably linked to the greater number of newborns with a gestational age between 28 and 37 weeks in the AKI group. In our study, there was a higher percentage of SGA infants in the SP group without AKI (23.9% vs. 10.8% in the AKI group). However, no significant differences were found in the percentage of IGR. These somewhat contradictory results are reflected in the literature: the meta-analysis of Liu Y et al. [15] found that neonates born to mothers with AKI had a lower birth weight than those born to mothers without AKI, and Cooke et al. [54], in a series of 26 patients with AKI from a cohort of 322 pregnant women, found that AKI had no substantial impact on perinatal prognosis. The risk of new-borns requiring admission to the neonatal ICU was 1.9 times higher in the group with AKI (OR 1.90; CI 95%: 1.11–3.28, *p* 0.002); however, perinatal mortality in the group of pregnant women with SP and AKI was 2.7%, significantly lower than that of the group with SP without AKI (7%). This result is in disagreement with the report of Liu Y et al. [15], who found that the risk of perinatal mortality in pregnant women with AKI was 3.39 times higher than that in pregnant women without AKI. Along this line, we must say that more studies are necessary to weigh the impact of AKI on neonates born to women with SP.

In the multivariate analysis performed to predict the risk of AKI, only a history of CKD, IVF, TMA (including HELLP syndrome), uric acid level and caesarean section were independently associated with the development of AKI in pregnant women with SP. CRF and pregestational proteinuria, which had a high magnitude of effect in the univariate analysis, lost their statistical significance in the multivariate analysis because both are related to CKD. When HELLP syndrome was considered in isolation outside of TMA, it also lost the ability to predict AKI, despite its important association with AKI in the univariate analysis. A likely explanation is the small sample size (*n* = 31). Uric acid, as a marker of renal risk, was maintained as a predictor of AKI, but the average haemoglobin, platelet and LDH values were not. The relationship of these analytical variables with TMA, defined as microangiopathic haemolytic anaemia and thrombocytopenia, probably justifies the loss of their statistical association with AKI, (Although in the opinion of the authors, due to the fact that a high IQ range is described, this value should be taken with caution despite its statistical association). Finally, caesarean section maintained its statistical significance, but the need for transfusion did not. The association of transfusion with TMA and postpartum haemorrhage, which is more common with caesarean delivery, may explain this result. Liu Y et al. [15] conducted a systematic review on the subject and found that pregnancy-associated AKI carries a significantly higher risk of caesarean section, postpartum haemorrhage, abruptio placentae, DIC and maternal death. At this point, we want to highlight that patients who resort to IVF are patients with more frequent previous pathologies that predispose to developing hypertensive pathology during pregnancy and endothelial lesion included at the renal level in early gestational ages [56]. Despite the fact that pregnant women with IVF have a higher rate of cesarean section, in our series we found an independent statistical association for the development of AKI.

At the same time, they found a significantly higher risk of perinatal mortality, prematurity and SGA. Regarding the renal prognosis, they observed a 2.4% incidence of the evolution to terminal renal failure with the need for renal replacement therapy.

The prognosis of AKI associated with preeclampsia is relatively good as long as there are no other associated complications, such as sepsis, DIC or severe bleeding, and most patients resume normal renal function postpartum [29,50]. Persistent renal dysfunction and the need for dialysis is more common in patients with previous CKD [20,57]. However, several authors have shown that pregnant women with preeclampsia have a higher risk of developing CKD than normotensive pregnant women [58,59]. Therefore, short- and long-term monitoring of renal function is necessary in women with a history of hypertensive disorders during pregnancy, childbirth and puerperium [60].

The study has limitations that are important to take into consideration when interpreting the data including a lack of other variables that were not the focus of this study, and that could provide additional information, such as ethnicity, body mass index or weight gain during pregnancy. In addition, we must clarify that since some study variables, such as the c-section, can occur before or after the appearance of AKI, as well as before or after the appearance of SP, the established causal associations must be analyzed with caution due to the fact that the temporality of the cause and effect is not fulfilled in all cases. In relation to the strengths of the study, although our results are not novel and support those obtained previously by the various studies already described in the discussion, with a large number of SP patients. All these patients were attended in a tertiary hospital in Madrid, one of the reference hospitals for obstetrics in Spain with more than 70,000 deliveries, attended over more than 11 years. Furthermore, our study analyses and compared the maternal and perinatal variables between AKI and No AKI groups.

## 5. Conclusions

Severe preeclampsia is constantly increasing in developing countries and in those patients, AKI is a common complication, especially among those with a history of CKD, those who became pregnant using assisted reproduction techniques and in cases of caesarean section. Among biochemical and haematological markers, the uric acid level prior to the development of AKI has a direct and significant correlation with the risk of AKI in patients with preeclampsia, as does the development of TMA. Therefore, the need for strict monitoring of renal function in cases of preeclampsia should be noted.

## Figures and Tables

**Table 1:** Comparative analysis of the distribution of pre-pregnancy variables in all patients with pre-eclampsia and in the study groups (AKI vs Non AKI). Cr: creatinine; Obesity: body mass index (BMI) ≥ 30 Kg/m^2^. Overweight BMI ≥ 25 Kg/m^2^.

	Total *n* = 303		AKI (*n* = 75)		No AKI (*n* = 228)		
	N; Mean ± S.T	%	Mean ± S.T	%	Mean ± S.T	%	*p*
Maternal age	33.94 ± 6.29		35.09 ± 6.99		33.56 ± 6.02		0.068
Nationality							0.570
Spanish	179	59.1		60		58.8	
South American	91	29.6		33.3		28.9	
African	14	4.5		4		4.8	
European not Spanish	14	4.5		2.7		5.3	
Other	5	1.5		0		2.2	
HT	49	16.2		18.7		15.4	0.499
Diabetes Mellitus	7	2.3		1.3		2.6	0.516
Hypothyroidism	30	9.9		9.3		10.1	0.480
BMI (Kg/m^2^)	26.23 ± 5.27		26.05 ± 5.44		26.29 ± 5.23		0.742
Obesity	66	24.0		21.1		25	0.510
Overweight	153	56.3		53.6		57.1	0.611
Autoimmune disease	7	2.3		4		1.8	0.261
CKD	24	7.9		17.3		4.8	0.001
CRF	5	1.7		5.3		0.4	0.004
Cr baseline serum (mg/dL)	0.64 ± 0.20		0.78 ± 0.31		0.60 ± 0.11		<0.001
Pregestational proteinuria	12	4		9.3		2.2	0.006
Number of pregnancies	2.01 ± 1.38		1.87 ± 1.31		2.06 ± 1.40		0.290
Nulliparity	153	50.5		54.7		49.1	0.405
Abortion History	107	35.3		26.7		38.2	0.071
Number of abortions	0.52 ± 0.87		0.44 ± 0.90		0.54 ± 0.81		0.352
History of preeclampsia	26	8.6		10.7		7.9	0.457
Family History of HT	116	38.3		39.7		39.4	0.957
Family history of preeclampsia	22	7.3		4		8.3	0.210

**Table 2:** Comparative analysis of the distribution of gestational variables in all patients with preeclampsia and in the study groups (AKI vs non AKI). UTI: urinary tract infection.

	Total *n* = 303		AKI (*n* = 75)		No AKI (*n* = 228)		
	*n*	%	*n*	%	*n*	%	*p*
IVF	63	20.8	24	32	39	17.1	0.006
Multiple pregnancy	55	18.2	19	25.3	36	15.8	0.063
HT before 20 weeks of follow-up	53	17.5	15	20	38	16.7	0.510
Gestational HT	78	25.7	14	18.7	64	28.1	0.106
Gestational Diabetes	30	9.9	8	10.7	22	9.6	0.798
Gestational hypothyroidism	33	10.9	11	14.7	22	9.6	0.480
Proteinuria < 20 weeks	12	4	7	9.3	5	2.2	0.006
UTI during pregnancy	97	32.1	25	33.3	72	31.6	0.778
Alphamethyldopa treatment	59	19.5	15	20	44	19.3	0.894
Calcium antagonist treatment	11	3.6	2	2.7	9	3.9	0.607
Labetalol treatment	40	13.2	18	23.5	22	17	0.758

**Table 3:** Comparative analysis of the distribution of the variables that refer to the time of diagnosis of severe preeclampsia in all the patients and in the study groups (AKI vs. non AKI). CrCl: Creatinine clearance.

	Total *n* = 303		AKI (*n* = 75)		No AKI (*n* = 228)		
	N; Mean ± SD	%	Mean ± SD	%	Mean ± SD	%	*p*
SBP mmHg	178.97 ± 16.52		179.04 ± 20.13		178.95 ± 15.20		0.971
DBP mmHg	103.84 ± 11.82		102.52 ± 13.81		104.28 ± 11.09		0.263
Gestational age at diagnosis	34.03 ± 4.37		33.31 ± 3.99		34.27 ± 4.46		0.098
Gestational age at diagnosis <28 weeks 28–36.6 weeks ≥37 weeks	25 175 103	8.3 57.8 34		8 72 20		8.3 53.1 36.6	0.010
Early initiation of SP	125	41.3		49.3		38.6	0.101
Puerperal PE	66	21.8		14.7		24.1	0.085
Proteinuria (g/24 h)	2.8 ± 2.78		3.18 ± 2.92		2.67 ± 2.72		0.170
Creatinine (mg/dL)	0.89 ± 0.53		1.53 ± 0.73		0.68 ± 0.14		<0.001
Urea (mg/dL)	37.88 ± 21.48		58.39 ± 25.83		29.68 ± 12.08		<0.001
CrCl (mL/min)	112.24 ± 42.65		81.64 ± 41.58		127.39 ± 34.37		<0.001
Uric acid (mg/dL)	6.80 ± 1.68		8.13 ± 1.54		6.36 ± 1.48		<0.001
Maximum uric acid level (mg/dL)	7.37 ± 1.74		8.87 ± 1.74		6.88 ± 1.45		<0.001
GOT (U/L)	110.28 ± 298.74		220.87 ± 488.14		60.880 ± 126.00		0.010
GPT (U/L)	68.50 ± 143.03		139.63 ± 244.96		45.10 ± 73.72		0.001
Platelets (×10^3^/µL)	165.25 ± 72.12		138.21 ± 78.15		174.25 ± 67.15		<0.001
LDH (U/L)	356.81 ± 340.91		534.15 ± 588.81		298.47 ± 166.71		0.001
Hemoglobin (g/dl)	10.66 ± 1.76		9.89 ± 2.05		10.91 ± 1.58		<0.001
Magnesium (mg/dL)	4.20 ± 2.59		5.28 ± 2.47		3.77 ± 2.52		<0.001
C3 level (mg/dL)	141.26 ± 39.46		131.59 ± 45.20		145.35 ± 36.15		0.025
C4 level (mg/dL)	26.34 ± 10.25		24.90 ± 11.15		26.96 ± 9.81		0.156
IgG level (mg/dL)	810.97 ± 283.01		756.81 ± 291.17		833.81 ± 277.23		0.056
IgA level (mg/dL)	202.78 ± 77.91		197.15 ± 80.34		205.16 ± 76.99		0.472
IgM level (mg/dL)	128.06 ± 63.05		119.10 ± 60.21		131.85 ± 64.02		0.157
Antiphospholipid 1st	9	3.4		4.4		3	0.575
ANA	16	6.7		5.6		7.1	0.670
antiDNA	4	1.7		1.4		1.8	0.853
Albumin (g/dL)	3.41 ± 0.68		3.21 ± 0.77		3.53 ± 0.60		0.004
Triglycerides (mg/dL)	194.30 ± 94.80		227.01 ± 95.35		180.80 ± 91.45		<0.001
Cholesterol (mg/dL)	247.51 ± 73.28		228.27 ± 64.76		255.25 ± 75.23		0.009
Magnesium sulfate treatment	208	68.6		82.7		64	0.003
Labetalol treatment	247	81.5		82.7		81.1	0.768
Treatment with hydralazine	98	32.3		8.9		23.4	0.435
Diuretic treatment	15	5		10.7		3.1	0.009
Oliguria	32	10.6		34.7		2.6	<0.001
HELLP syndrome	31	10.2		25.3		5.3	<0.001
TMA (includes HELLP)	35	11.6		29.3		5.7	<0.001
Eclampsia	10	3.3		0		4.4	0.065

**Table 4:** Comparative analysis of the distribution of intrapartum and immediate postpartum variables in all patients with pre-eclampsia and in the study groups (AKI vs non AKI). ARB: angiotensine receptor blockers.

	Total *n* = 303		AKI (*n* = 75)		Non AKI (*n* = 228)		
	Mean ± SD	%	Mean ± SD	%	Mean ± SD	%	*p*
Delivery follow-up	34.46 ± 3.86		33.80 ± 3.60		34.68 ± 3.92		0.089
Gestational age at diagnosis <28 weeks 28–36.6 weeks ≥37 weeks	16 178 109	5.3 58.7 36		5.3 73.3 21.3		5.3 53.9 40.8	0.008
Birth initiation method: Spontaneous Induced Elective caesarean section	25 135 143	8.3 44.6 47.2		4 38.7 57.3		9.6 46.5 43.9	0.077
Completion of delivery: Vaginal Caesarean	107 196	35.3 64.7		17.3 82.7		41.2 58.8	<0.001
HT at delivery	260	85.8		86.7		85.5	0.806
Transfusion	30	9.9		6.9		3	<0.001
Postpartum Labetalol	292	96.4		96		96.5	0.844
Postpartum hydralazine	147	48.5		56		46.3	0.143
Postpartum Enalapril	275	90.8		80		94.3	<0.001
ARB II postpartum	17	5.6		8.1		4.8	0.291
Postpartum calcium antagonists	202	66.7		66		70.2	0.031
Postpartum furosemide	102	33.7		44		30.3	0.082

**Table 5:** Comparative analysis of the distribution of perinatal variables in all patients with pre-eclampsia and in the study groups (AKI vs non AKI). SGA: small for their gestational age. Newborn: NB.

	Total *n* = 303/351		AKI (*n* = 75)		No AKI (*n* = 228)		
	Mean ± SD	%	Mean ± SD	%	Mean ± SD	%	*p*
Follow-up at birth	34.46 ± 3.86		33.80 ± 3.60		34.68 ± 3.92		0.089
Lung maturation	146	48.2		60		44.3	0.018
Intrauterine growth restriction (IUGR)	71	20.2		18.7		25	0.261
SGA 1st Newborn (non IUGR)	62	17.7		10.8		23.9	0.036
Sex 1st Newborn Male Female	138 165	45.5 54.5		41.3 58.7		46.9 53.1	0.399
Sex 2nd Newborn Male Female	20 28	41.7 58.3		41.2 58.8		41.9 58.1	0.959
Weight 1st Newborn (grams)	2.128.24 ± 864.29		2.023.81 ± 851.33		2.162.59 ± 867.61		0.228
Weight 2nd Newborn (grams)	1.919.86 ± 504.70		2.052.22 ± 433.28		1.845.41 ± 532.71		0.167
Apgar Test value 1st min	7.52 ± 1.76		6.96 ± 1.82		7.73 ± 1.67		0.002
Apgar Test Value 1st min 2nd NB	7.94 ± 1.23		7.72 ± 1.36		8.06 ± 1.16		0.355
Apgar Test Value 5 min 1st NB	8.44 ± 1.70		8.5 ± 1.43		9.02 ± 1.18		0.006
Apgar test value 5 min 2nd NB	9.16 ± 0.86		9.11 ± 0.90		9.19 ± 0.85		0.768
1st NB cord pH	7.24 ± 0.09		7.23 ± 0.11		7.25 ± 0.08		0.310
2nd NB cord pH	7.29 ± 0.08		7.28 ± 0.11		7.29 ± 0.07		0.513
Exitus perinatal	18	5.1		2.7		7	0.165
Admission to Neonatal Intensive Care Unit	96	27.4		42.7		28.1	0.018
Cause of admission Heart disease Distres Intubation difficulty Prematurity IUGR	15 37 10 13 21	15.6 38.5 10.4 13.5 21.9		9.4 50.0 15.6 12.5 12.5		18.8 32.8 7.8 14.1 26.6	0.20

**Table 6:** Comparative analysis of the distribution of variables at hospital discharge in all patients with pre-eclampsia and in the study groups (AKI vs non AKI).

	Total *n* = 303		AKI (*n* = 75)		No AKI (*n* = 228)		
	Mean ± SD	%	Mean ± SD	%	Mean ± SD	%	*p*
HT	288	95		86.7		97.8	<0.001
Number of Antihypertensive treatment							
0 1 2 ≥3	16 130 132 25	5.3 43.9 43.6 8.3		13.3 46.7 33.3 6.7		2.6 41.7 46.9 8.8	0.002
Proteinuria	219	72.3		71.6		75.1	0.552
Creatinine (mg/dL)	0.68 ± 0.26		0.88 ± 0.42		0.62 ± 0.12		<0.001
Urea (mg/dL)	33.15 ± 13.59		41.18 ± 18.73		29.90 ± 9.07		<0.001
Ccr (mL/min)	123.49 ± 34.58		104.35 ± 37.38		130.71 ± 30.61		<0.001
Uric acid (mg/dL)	5.73 ± 1.44		6.21 ± 1.72		5.58 ± 1.30		0.005
Proteinuria (g/24 h)	1.04 ± 1.17		1.36 ± 1.51		0.93 ± 1.02		0.026
GOT (U/L)	31.87 ± 23.60		36.22 ± 27.54		29.95 ± 21.46		0.075
GPT (U/L)	30.17 ± 25.08		39.56 ± 36.01		27.08 ± 19.38		0.005
Platelets at discharge (×10^3^/µL)	288.45 ± 101.82		294.00 ± 115.21		286.02 ± 97.18		0.574
LDH (U/L)	245.75 ± 82.36		268.36 ± 122.88		238.28 ± 62.17		0.045
Haemoglobin (g/dL)	11.22 ± 1.45		10.87 ± 1.48		11.33 ± 1.43		0.016

**Table 7:** Comparative analysis of the distribution of variables at discharge from the risk puerperium consultation in all patients with pre-eclampsia and in the study groups (AKI vs non AKI).

	Total *n* = 303		AKI (*n* = 75)		No AKI (*n* = 228)		
	Mean ± SD	%	Mean ± SD	%	Mean ± SD	%	*p*
Persistent hypertension	72	23.8		24.3		25.8	0.797
Persistent proteinuria	31	11.1		21.4		7.6	0.001
Renal insufficiency	7	2.3		9.9		0	<0.001
Proteinuria (mg/mg)	0.19 ± 0.47		0.38 ± 0.88		0.13 ± 0.21		0.032
Serum creatinine (mg/dL)	0.69 ± 0.21		0.85 ± 0.35		0.65 ± 0.11		<0.001
CrCl (mL/min)	115.17 ± 29.8		99.61 ± 27.57		121.68 ± 28.37		<0.001
Evolution Referral to family doctor Referral to Nephrology Loss of follow-up	203 72 28	67 23.8 9.2		64 29.3 6.7		68 21.9 10.1	0.341

## Data Availability

Data from this study are available at the obstetrics service of the Hospital General Universitario Gregorio Marañón in Madrid and will be made available upon request.
